# Cytotoxicity of Triorganophosphinegold(I) Complexes of Thiobenzoate

**DOI:** 10.1155/MBD.2001.79

**Published:** 2001

**Authors:** Beverly R. Vincent, David J. Clarke, Douglas R. Smyth, Dick de Vos, Edward R. T. Tiekink

**Affiliations:** 1 Molecular Structure Corporation 9009 New Trails Drive The Woodlands Texas 77381-5290 USA; 2 Department of Chemistry The University of Adelaide 5005 Australia; 3 Medical Department Pharmachemie B.V. Haarlem NL-2003 The Netherlands

## Abstract

The preparation and characterization of two triorganophosphinegold(I) complexes containing the anion derived from thiobenzoic acid are described. The cytotoxicity of these complexes has been investigated along with that of triphenylphosphinegold(I) mercaptopurinate, a known anti-tumor compound, against a variety of human cell lines. The complexes showed moderate to high cytotoxicity (ID_50_ 250 – 2500 ng/ml).


### CYTOTOXICITY OF TRIORGANOPHOSPHINEGOLD(I) COMPLEXES OF THIOBENZOATE

Beverly R. Vincent,[1] David J. Clarke,[2] Douglas R. Smyth,[2] Dick de Vos,[3] and Edward R. T. Tiekink[2]*

[1] Molecular Structure Corporation, 9009 New Trails Drive, The Woodlands, Texas 77381-5290, USA
[2] Department of Chemistry, The University of Adelaide, Australia 5005
[3] Medical Department, Pharmachemie B.V., NL-2003 Haarlem, The Netherlands
e-mail: edward.tiekink@adelaide.edu.au

**Abstract**
The preparation and characterization of two triorganophosphinegold(I) complexes containing the anion derived from thiobenzoic acid are described. The cytotoxicity of these complexes has been investigated along with that of triphenylphosphinegold(I) mercaptopurinate, a known anti-tumor compound, against a variety of human cell lines. The complexes showed moderate to high cytotoxicity ($ID_{50}$ 250 – 2500 ng/ml).

**Introduction**
Auranofin (triethylphosphinegold(I) tetraacetatothioglucose), featuring a linear P-Au-S arrangement, is used clinically in the treatment of rheumatoid arthritis [1 – 3]. Auranofin and analogues also possess cytotoxicity and anti-tumor activity [4 – 9]. Consequently, research into the potential anti-tumor activity of systems related to auranofin has attracted considerable attention [9]. A particularly interesting series of complexes are those containing biologically active thiols such as 6-mercaptopurine. Previous studies have shown that potency is found in cisplatin-resistant cell lines and that activity is maintained *in vivo* [10 – 14]. This contribution reports the cytotoxicity of two complexes, [$Cy_3PAu(SC(O)Ph)$] (**1**) and [$Ph_3PAu(SC(O)Ph)$] (**2**), containing the anion derived from thiobenzoic acid against a panel of seven human cancer lines. For comparison, the cytotoxicity of [$Ph_3PAu$(6-MP)], isolated as an ethanol solvate and a known anti-tumor compound [12], is also reported.

**Experimental**
*General:* IR spectra were recorded as KBr disks on a Perkin-Elmer FT-IR spectrophotometer. $^1H$, and $^{13}C$ spectra were measured on a Varian Gemini-200 FT/spectrometer operating at 199.953 and 50.283 MHz, respectively, and $^{31}P$ NMR were measured on a Varian Gemini 2000 spectrometer operating at 121.501 MHz. Spectra were referenced to TMS or solvent where appropriate. Analytical grade solvents were used without further purification. $HAuCl_4$ [15] and $R_3PAuCl$ [16] were prepared according to the literature procedures. Thiobenzoic acid (Aldrich) was used without further purification.
*Synthesis:* The preparations of the colorless gold complexes were in accord with established literature methods [11, 12, 17].

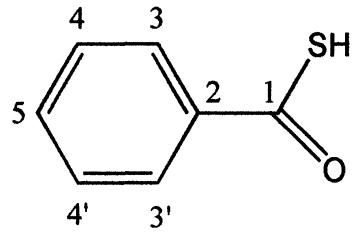

**Figure 1.** Numbering scheme for thiobenzoic acid.

[$Cy_3PAu(SC(O)Ph)$] (**1**) Yield 87 %, m. p. 190 – 191 °C. IR: ν(C=O) 1620 $cm^{-1}$. NMR: $^1H$ δ 8.15 (br, 2H, *H*3; see Figure 1 for atom numbering scheme); 7.39 (br, 2H, *H*4); 7.48 (br, 1H, *H*5); 2.21 – 1.31 ppm (Cy-*H*). $^{13}C$ δ 201.9, 142.9, 129.6, 128.4, 131.5 (C1 – C5); 34.2 (d, 27.9 Hz, Cα), 27.2 (d, 11.9 Hz, Cβ); 31.5 (Cγ), 26.6 ppm (Cδ). $^{31}P$ δ 57.0 ppm.

[$Ph_3PAu(SC(O)Ph)$] (**2**) Yield 69 %, m. p. 148 – 150 °C. IR: ν(C=O) 1611 $cm^{-1}$. NMR: $^1H$ δ 8.13 (br, 2H, *H*3); 7.65 – 7.34 ppm (m, 18H, *H*4, *H*5 and Ph-*H*). $^{13}C$ δ 201.1, 142.0, 128.4, 127.8, 131.7 (C1 –

C5); 129.4 (d, 58.4 Hz, Cα), 134.3 (d, 13.8 Hz, Cβ); 129.2 (d, 11.4 Hz, Cγ), 131.7 ppm (Cδ). $^{31}P$ δ 38.5 ppm.

[$Ph_3PAu$(6-MP)].EtOH (**3**) Yield 64 %, m. p. 250 – 252 °C (dec.), Lit. [17] 254 – 255 °C.

*Biological evaluations:* The cytotoxicity of **1** – **3** and a series of common anti-tumor agents was evaluated in the following human tumor cell lines: MCF7 (breast cancer), EVSA-T (breast cancer), WIDR (colon cancer), IGROV (ovarian cancer), M19 MEL (melanoma), A498 (renal cancer) and H226 (non-small cell lung cancer). The MCF7 cell line is estrogen receptor (ER)+/progesterone receptor (PgR)+ and the cell line EVSA-T is (ER)-/(PgR)-. Prior to the experiments a mycoplasma test was carried out on all cell lines and found to be negative. All cell lines were maintained in a continuous logarithmic culture in RPMI 1640 medium with Hepes and phenol red. The medium was supplemented with 10% FCS, penicillin 100 IU/ml and streptomycin 100 μg/ml. The cells were mildly trypsinized for passage and for use in the experiments.

Chemicals. RPMI and FCS were obtained from Life technologies (Paisley, Scotland). SRB, DMSO, penicillin and streptomycin were obtained from Sigma (St Louis MO, USA), trichloroacetic acid and acetic acid from Baker BV (Deventer, NL) and PBS from NPBI BV (Emmer-Compascuum, NL).

Experimental procedures. The test and reference compounds were dissolved to a concentration of 250000 ng/ml in full medium, by 20-fold dilution of a stock solution, which contained 1 mg of compound/200 μl. The compounds were dissolved in DMSO. Cytotoxicity was estimated by the microcultuer sulforhodamine B (SRB) test [18].

Cytotoxicity. The experiment was started on day 0 when 150 μl of trypsinized tumor cells (1500 – 2000 cells/well) were plated in 96-wells flatbottom microtiter plates (falcon 3072, BD). The plates were preincubated 48 h at 37 °C, 8.5 % $CO_2$ to allow the cells to adhere. On day 2, a 3-fold dilution sequence of ten steps was made in full medium, starting with the 250000 ng/ml stock solution. Every dilution was used in quadruplicate by adding 50 μl to a column of four wells. This results in a highest concentration of 62500 ng/ml present in column 12. Column 2 was used for the blank. To column 1, PBS was added to diminish interfering evaporation. On day 7 the incubation was terminated by washing the plate twice with PBS. Subsequently, the cells were fixed with 10 % trichloroacetic acid in PBS and placed at 4 °C for 1 h. After 5 washings with tap water, the cells were stained for at least 15 min with 0.4 % SRB dissolved in 1 % acetic acid. After staining the cells were washed with 1 % acetic acid to remove the unbound stain. The plates were air-dried and the bound stain was dissolved in 150 μl 10 mM Tris-base. The absorbance was read at 540 nm using an automated microplate reader (Labsystems Multiskan MS). Data were for construction of concentration-response curves and determination of the $ID_{50}$ value by use of Deltasoft 3 software.

*Crystallography:* Crystals of [$Cy_3PAu$(SC(O)Ph)] (**1**) and [$Ph_3PAu$(6-MP)].EtOH (**3**) were obtained from the slow evaporation of ethanol/chloroform solution of the respective compound. Relevant crystallographic data is collected in Table 1. Data were collected at 173 K on a Rigaku/MSC Mercury CCD area detector and the weighting scheme was of the form $1/[\sigma^2(F_o)]$. For **3**, H atoms involved in intermolecular hydrogen bonding were located from a difference map and included in the model.

**Table 1.** Crystal data for [$Cy_3PAu$(SC(O)Ph)] (**1**) and [$Ph_3PAu$(6-MP)].EtOH (**3**)

| | **1** | **3** |
|---|---|---|
| Formula | $C_{25}H_{38}AuOPS$ | $C_{25}H_{24}AuN_4OPS$ |
| Formula weight | 614.6 | 656.5 |
| Crystal size, mm | 0.10 x 0.30 x 0.40 | 0.20 x 0.20 x 0.20 |
| Crystal system | triclinic | monoclinic |
| Space group | *P*barl | $P2_1/n$ |
| *a*, Å | 9.091(5) | 8.7178(2) |
| *b*, Å | 11.441(5) | 27.539(1) |
| *c*, Å | 12.559(4) | 10.6542(7) |
| *α* ° | 73.95(2) | 90 |
| *β*, ° | 83.98(5) | 107.84(1) |
| *γ* ° | 88.63(11) | 90 |
| *V*, Å 3 | 1248.4(9) | 2434.8(2) |
| *Z* | 2 | 4 |
| μ(Mo-Kα), cm-1 | 60.74 | 62.40 |
| $D_{calcd}$, g cm-3 | 1.635 | 1.791 |
| *F*(000) | 612 | 1280 |
| $\theta_{max}$, ° | 30.9 | 26.6 |
| Reflns meas, unique, $R_{int}$ | 14092, 5953, 0.023 | 24146, 5113, 0.046 |
| Reflns with $I \geq 3.0\sigma(I)$ | 5314 | 3507 |
| Refined parameters | 262 | 298 |
| *R*(*F*), *wR*(*F*) | 0.021, 0.025 | 0.025, 0.024 |
| ρ, e Å-3 | 0.73 | 2.11 |
| Programs used | CrystalClear [19], teXsan [20], | DIRDIF [21], ORTEP [22] |
| Deposition number | CCDC 158867 | CCDC 158866 |

**Results and Discussion**

*Characterization of 1 and 2*: The major feature of interest in the solid state IR spectra (KBr disks) related to the shift of ν(C=O) from 1681 $cm^{-1}$ in the acid to 1620 (**1**) and 1611 (**2**) $cm^{-1}$ for the complexes. The $^{1}H$ and $^{13}C$ resonances were assigned on the basis of the interpretation of their Heteronuclear Multiple Quantum Coherence (HMQC) and Heteronuclear Multiple Bond Connectivity (HMBC) spectra. Upon complexation, resonances due to H3 are shifted downfield compared to that for the uncoordinated acid, *i.e.* δ 7.90 ppm. By contrast, for **1** resonances due to H4 and H5 are shifted marginally upfield compared to the free ligand (*i.e.* δ 7.47 and 7.61 ppm, respectively); these resonances were obscured for **2**. Upon complexation, there is no significant shift in the $^{13}C$ resonances ascribed to C3-C5. However, the respective resonances due to C1 and C2 are shifted downfield by approximately 10 and 6 ppm, respectively compared to the free ligand. Small shifts in the $^{31}P$ resonances, compared to the $R_3PAuCl$ precursors, provide further evidence for complexation. Crystals of **1** were obtained allowing for a full structure determination by X-ray crystallographic methods.

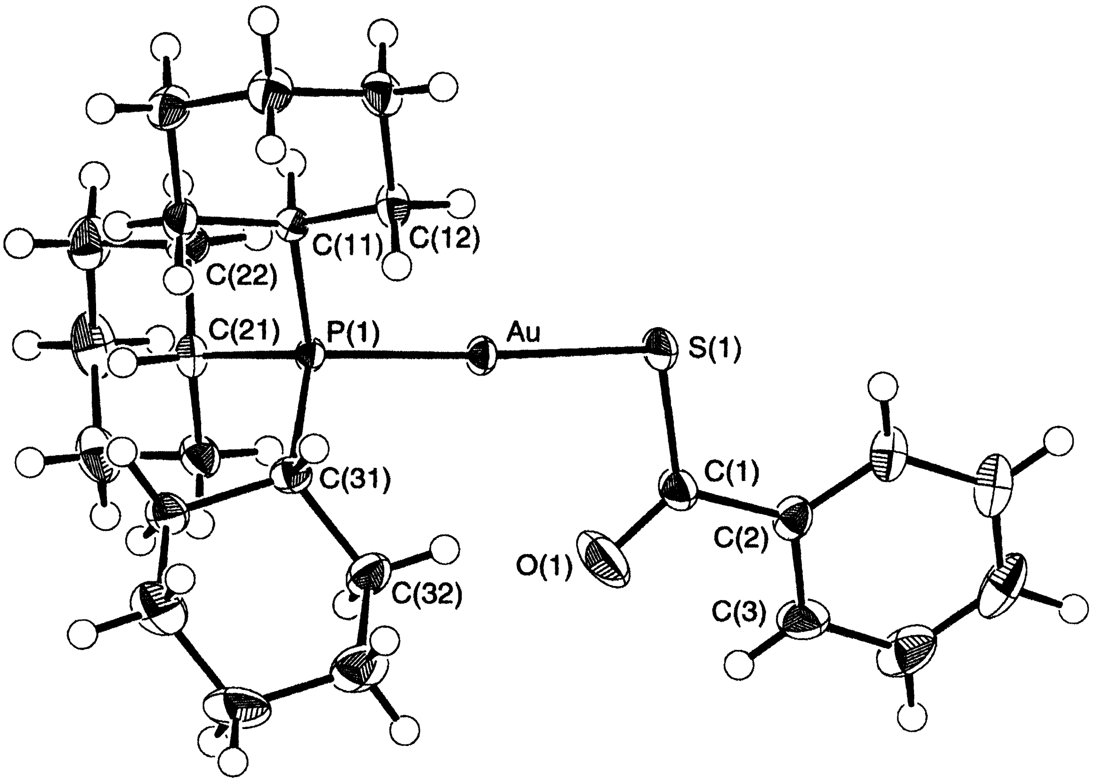

**Figure 2.** Molecular structure (50% probability ellipsoids) of $[Cy_3PAu(SC(O)Ph)]$ (**1**). Selected bond distances and angles: Au-S(1) 2.325(2), Au-P(1) 2.279(1), S(1)-C(1) 1.776(4), O(1)-C(1) 1.217(4) Å; S(1)-Au-P(1) 176.46(3), Au-S(1)-C(1) 102.24(11), S(1)-C(1)-O(1) 123.1(3)°.

The molecular structure of **1** is shown in Figure 2 and selected geometric parameters are collected in the Figure caption. The gold atom exists in the expected linear geometry defined by the S and P donor atoms. As is usual for complexes of this type, the Au-S distance is longer than the Au-P distance. This pattern of bond distance variation coupled with length of the C-S distance indicates that the ligand is functioning as a thiolate. There is a small deviation from the ideal linear geometry may be due to the close approach of the carbonyl oxygen atom. The Au...O separation is 3.197(3) Å, a distance resembling those found in related systems [23, 24]. Although full details are not reported here owing to high residual electron density peaks, a preliminary investigation of the structure of **2** revealed a similar coordination pattern as that reported for **1**. The molecular structure of **3**, $[Ph_3PAu(6\text{-}MP)].EtOH$, has also been determined.

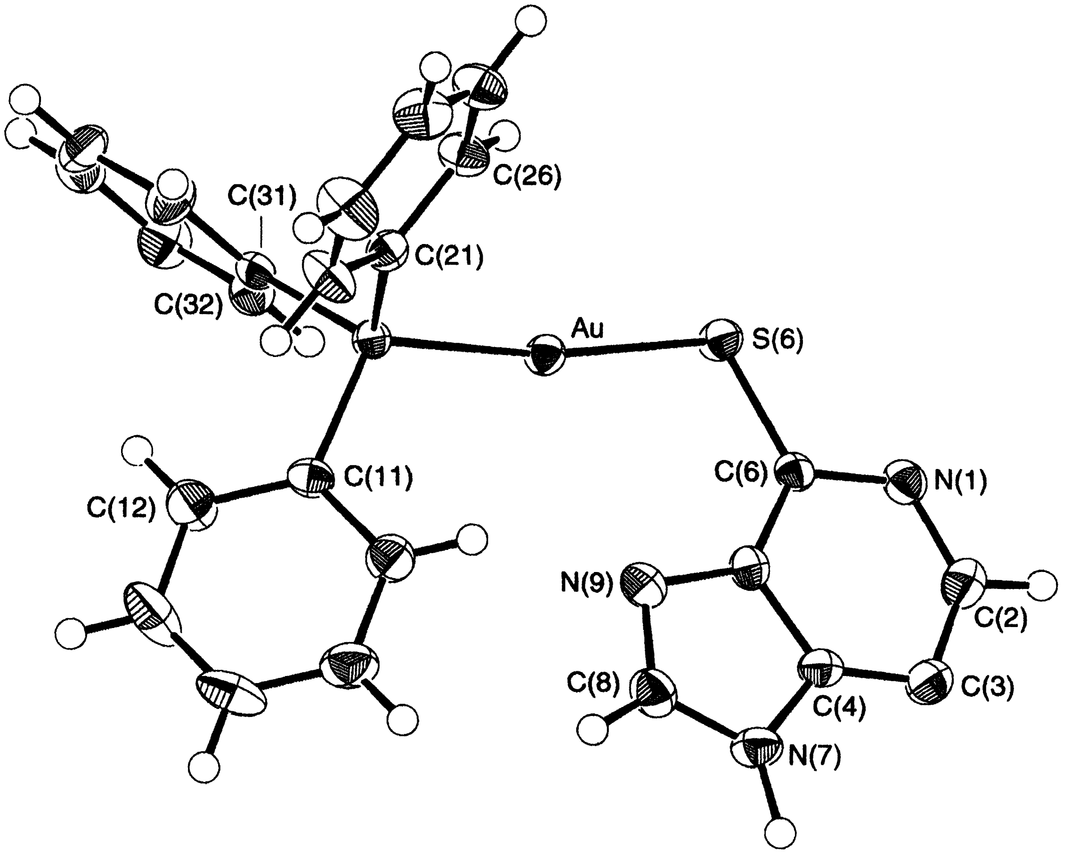

**Figure 3.** Molecular structure (50% probability ellipsoids) of [$Ph_3PAu$(6-MP)].EtOH (**3**). Selected bond distances and angles: Au-S(6) 2.311(2), Au-P(1) 2.258(1), S(6)-C(6) 1.739(5) Å; S(6)-Au-P(1) 171.17(4), Au-S(6)-C(6) 105.6(2)°.

*Characterization of 3*: [$Ph_3PAu$(6-MP)].EtOH (**3**) was also prepared in order to provide another reference compound for the cytotoxicity screening as **3** has been the subject of several investigations in this context [10 - 13]. During purification of the compound crystals were isolated and found to represent a second polymorphic form for this compound. The molecular structure, Figure 3, is in essential agreement with that found in the triclinic modification [17].

The interatomic parameters in the two polymorphs are equal within experimental error. By contrast, there are some small but significant differences in bond angles so that in the present structure the S(6)-Au-P(1) and Au-P(1)-C(11) bond angles of 171.17(4)° and 114.4(2)°, respectively may be compared to 173.71(6)° and 114.2(2)° in the triclinic polymorph [17]. The Au/S(1)/C(6)/C(5) torsion angle of 3.6(5)° facilitates the close approach of the N(7) atom to gold such that the separation between them is 2.889(4) Å. This distance is within the sum of the van der Waals radii for these atoms of 3.25 Å [25]. The crystal lattice of **3** features intermolecular hydrogen-bonding interactions as illustrated in Figure 4.

As can be seen from Figure 4, translationally related molecules are linked via ethanol molecules of crystallization. The ethanol molecule forms a donor interaction via O<u>H</u>(4) to a N(1)' atom so that H(4)...N(1)' is 1.82 Å, O(4)...N(1)' is 2.750(6) Å and the angle subtended at H(4) is 168°; symmetry operation: $x$-1, $y$, $z$. An acceptor interaction is also formed to N-<u>H</u>(7) with H(7)...O(4) 1.69 Å, N(7)...O(4) 2.732(5) Å and N(7)-H(7)...O(4) of 172°. In this way, molecules are linked to form chains aligned along the crystallographic $a$-direction.

*Cytotoxicity*: The new compounds **1** and **2**, the known anti-tumor compound **3** and several standards were screened for cytotoxicity in a panel of human cancer lines as detailed in the Experimental section. Results of the screen are summarized in Table 2.

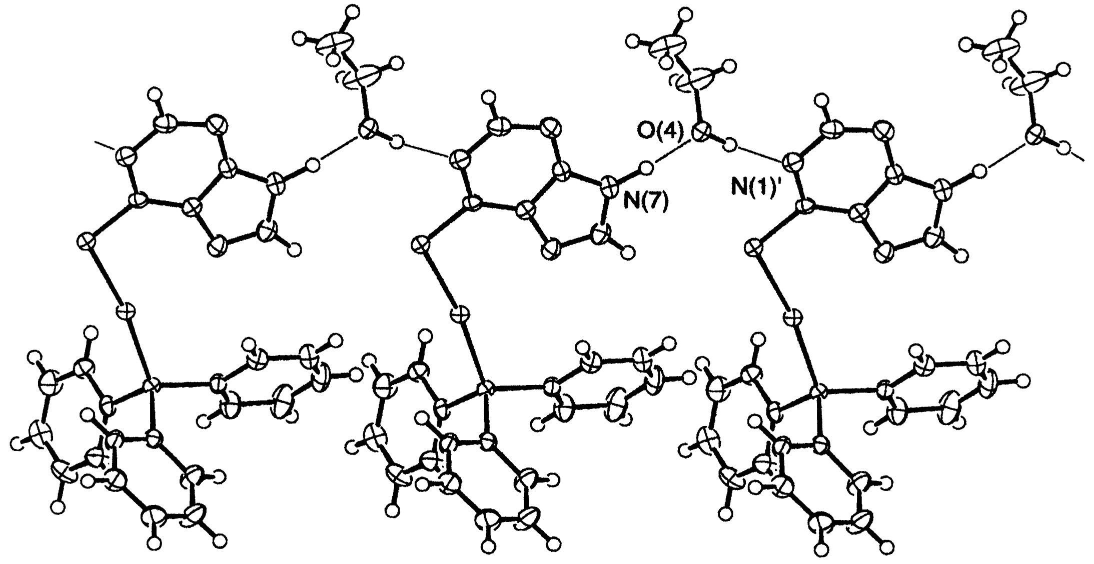

**Figure 4.** Molecular aggregation in the structure of [$Ph_3PAu$(6-MP)].EtOH (**3**).

**Table 2.** Cytotoxicity data ($ID_{50}$, ng/ml) for [$Cy_3PAu$(SC(O)Ph)] (**1**), [$Ph_3PAu$(SC(O)Ph)] (**2**), [$Ph_3PAu$(6-MP)].EtOH (**3**) and established cytotoxic agents[a]

| Compound | A498 | EVSA-T | H226 | IGROV | M19 | MCF-7 | WIDR |
|---|---|---|---|---|---|---|---|
| (1) | 4358 | 3319 | 6293 | 2916 | 2805 | 2805 | 5135 |
| (2) | 954 | 826 | 1124 | 324 | 1031 | 1010 | 942 |
| (3) | 1236 | 456 | 738 | 347 | 915 | 943 | 1200 |
| cisplatin | 2253 | 422 | 3269 | 169 | 558 | 699 | 967 |
| DOXb | 90 | 8 | 199 | 60 | 16 | 10 | 11 |
| 5-FUb | 143 | 475 | 340 | 297 | 442 | 750 | 225 |
| MTXb | 37 | 5 | 2287 | 7 | 23 | 18 | <3.2 |
| ETO[b] | 1314 | 317 | 3934 | 580 | 505 | 2594 | 150 |
| TAXb | <3.2 | <3.2 | <3.2 | <3.2 | <3.2 | <3.2 | <3.2 |

a See Experimental for details of cell lines.

b Abbreviations: DOX doxorubicin, 5-FU 5-fluorouracil, MTX methotrexate, ETO etoposide, TAX taxol.

Of the thiobenzoate compounds **1** and **2**, the triphenylphosphine compound was uniformally more active than the cyclohexylphosphine. This result is in contrast to the conclusions of earlier studies on related systems that suggest that compounds containing cyclohexylphosphine are the more potent [13]. The known anti-tumour compound **3** had comparable activity to that of **2**. The two triphenylphosphine derivatives were more active than cisplatin in the A498 and H226 cell lines and had comparable or reduced potency in the remaining cell lines. Thus, it may be concluded that while the gold-containing compounds examined had good to high cytotoxcity, they were not as active as cisplatin in all human cell lines.

**Acknowledgments**
The Australian Research Council is thanked for support.

**References**
[1] R.V. Parish, *Interdiscip. Sci. Rev.*, 1992, **17**, 221.
[2] P.J. Sadler and R.E. Sue, *Metal-Based Drugs*, 1994, **1**, 107.
[3] Tiekink ERT, Whitehouse MW. In: G. Berthon, ed. Handbook of Metal-Ligand Interactions in Biological Fluids, Marcel Dekker Inc, New York, Volume 2, 1995, 1266.

[4] T.M. Simon, D.H. Kunishima, G.J. Vibert and A. Lorber, *Cancer*, 1979, **44**, 1965.
[5] A.E. Finkelstein, O.R. Burrone, D.T. Walt and A. Misher, *J. Rheumatol.*, 1977, **4**, 245.
[6] T.M. Simon, D.H. Kunishima, G.J. Vibert and A. Lorber, *J. Rheumatal. (suppl 5)*, 1979, **6**, 91.
[7] T.M. Simon, D.H. Kunishima, G.J. Vibert and A. Lorber, *Can. Res.*, 1981, **41**, 94.
[8] C.K. Mirabelli, R.K. Johnson, C.M. Sung, L.F. Faucette, K. Muirhead and S.T. Crooke, *Can. Res.*, 1985, **45**, 32.
[9] E.R.T. Tiekink, *Crit. Rev. Oncol. & Haematol.*, 2001, in press.
[10] E.R.T. Tiekink, In Ph. Collery, J. Corbella, J.L. Domingo, J.C. Etienne and J.M. Llobet, eds. Metal Ions in Biology and Medicine. 1996, **4**, 693.
[11] E.R.T. Tiekink, P.D. Cookson, B.M. Linahan and L.K. Webster, *Metal-Based Drugs*, 1994, **1**, 299.
[12] L.K. Webster, S. Rainone, E. Horn and E.R.T. Tiekink, *Metal-Based Drugs*, 1996, **3**, 63.
[13] D. Crump, G. Siasios and E.R.T. Tiekink, *Metal-Based Drugs*, 1999, **6**, 361.
[14] D. de Vos, P. Clements, S.M. Pyke, D.R. Smyth and E.R.T. Tiekink, *Metal-Based Drugs*, 1999, **6**, 31.
[15] B.P. Block, *Inorg. Synth.*, 1953, **4**, 14.
[16] A.K. Al-Saady, C.A. McAuliffe, R.V. Parish and J.A. Sandbank, *Inorg. Synth.*, 1985, **23**, 191.
[17] P.D. Cookson, E.R.T. Tiekink and M.W. Whitehouse, *Aust. J. Chem.*, 1994, **47**, 577.
[18] Y.P. Keepers, P.E. Pizao, G.J. Peters, J. van Ark-Otte, B. Winograd and H.M. Pinedo, *Eur. J. Cancer*, 1991, **27**, 897.
[19] CrystalClear, Software for Automated X-ray Imaging Systems, Molecular Structure Corp. (The Woodlands, TX) and Rigaku Corp. (Tokyo).
[20] *teXsan, Single Crystal Structure Analysis Software*, Version 1.04 (1997), Molecular Structure Corporation, The Woodlands, TX, USA.
[21] P.T. Beurskens, G. Admiraal, G. Beurskens, W.P. Bosman, S. Garcia-Granda, R.O. Gould, J.M.M. Smits and C. Smykalla, The DIRDIF program system, Technical Report of the Crystallography Laboratory, University of Nijmegen, The Netherlands (1992).
[22] C.K. Johnson, ORTEP. Report ORNL-5138 (1976), Oak Ridge National Laboratory, TN, USA.
[23] G. Siasios and E.R.T. Tiekink, *Z. Kristallogr.*, 1993, **204**, 95.
[24] G. Siasios and E.R.T. Tiekink, *Z. Kristallogr.*, 1993, **205**, 261.
[25] A. Bondi, *J. Phys. Chem.*, 1964, **68**, 441.

**Received: March 6, 2001 – Accepted: March 23, 2001 – Accepted in publishable format: March 26, 2001**

